# Supplementary material for: Capsid-like particles decorated with the SARS-CoV-2 receptor-binding domain elicit strong virus neutralization activity
Source: Nat Commun. 2021 Jan 12;12:324. doi: 10.1038/s41467-020-20251-8 (PMC7804149; doi:10.1038/s41467-020-20251-8)
Supplement: Supplementary file 1 — Supplementary Information [file 41467_2020_20251_MOESM1_ESM.pdf]

Supplementary figures

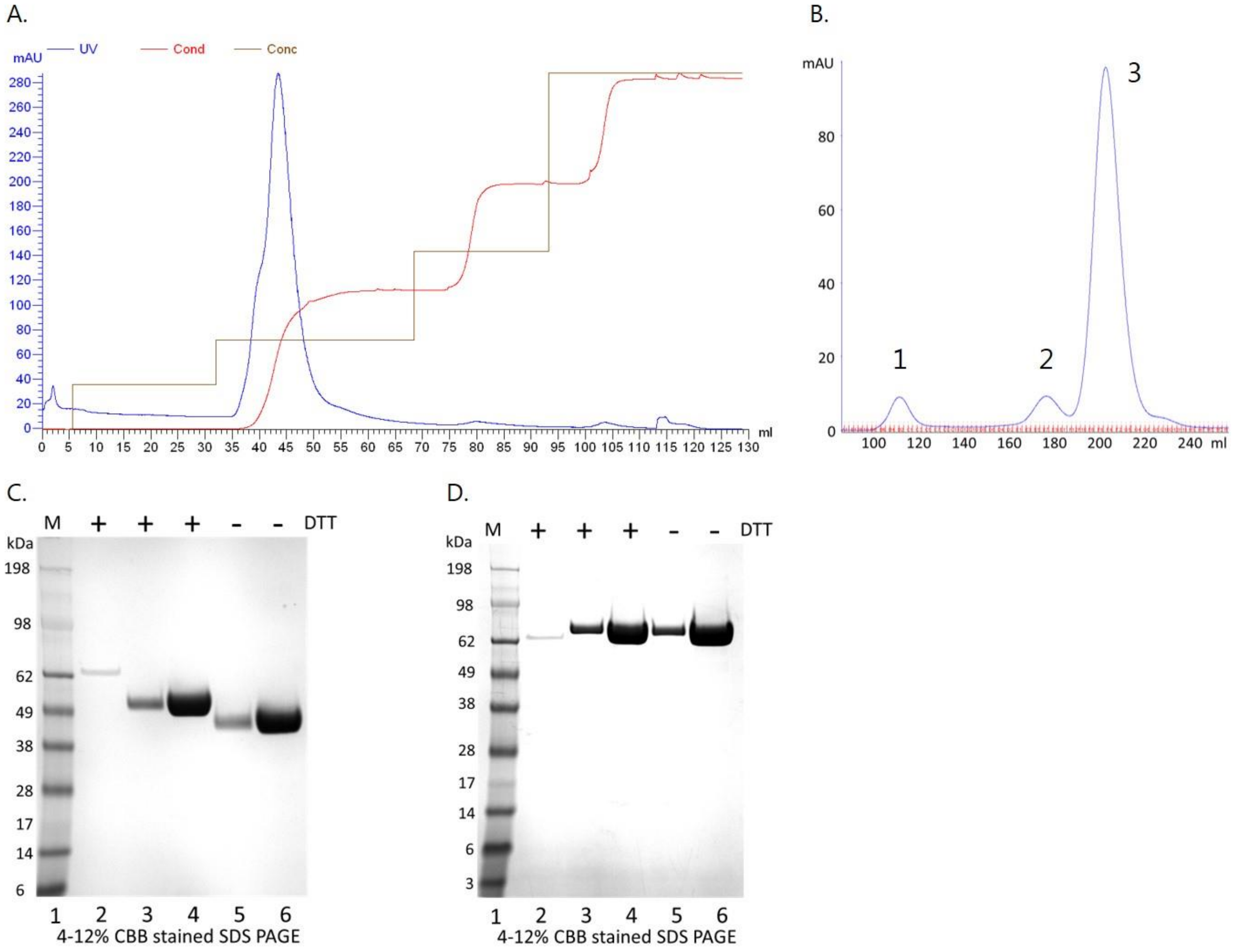

Supplementary figure 1: ExpreS[2] expression and purification of RBDc and ACE2 proteins. (A) Capture Select C tag elution chromatogram of the RBDc protein. The RBDc protein from 450 ml supernatant was loaded on and eluted from 4 ml Capture Select C tag resin by stepwise elution with MgCl2. Optical density at OD280nm (blue), programmed gradient of MgCl2 (brown). The bulk of protein elutes at 500mM $MgCl_2$. (B) Preparative size exclusion chromatography of the RBDc protein using a 120 ml Superdex 200 pg column (Cytiva). The RBDc protein mainly behaves as a monomer in solution (peak 3) with smaller leading peaks (peak 1 and 2). (C, D) Coomassie stained SDS-PAGE analysis of RBDc (C) and ACE2 protein (D). Purification and SDS gels were repeated at least 5 times for each, and all showed comparable results. Loading pattern is identical in C & D. Lane 1: marker, Lane 2: 0.1 µg BSA, Lanes 3 and 5: 1 µg protein, lanes 4 and 6: 10 µg protein. Lane 1-4: reduced, lanes 5-6: unreduced.

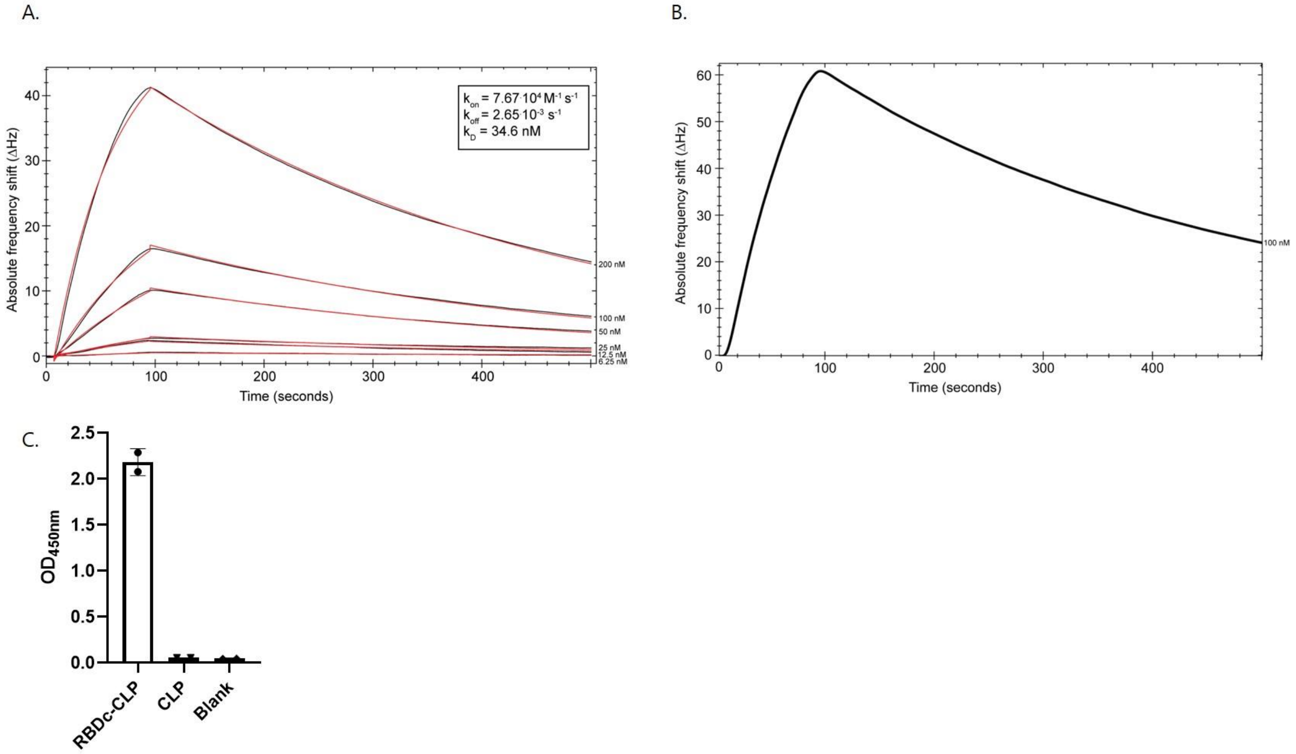

Supplementary figure 2: RBDc and RBDc-CLP bind to ACE2 *in vitro*. (A) Real time binding (black curves) of ExpreS^2 ACE2 to immobilized RBDc on the chip surface. Red curves show theoretical curves obtained using a 1:1 simple binding model. Analyte concentrations are shown to the right and k_{on}, k_{off} and k_D are boxed. (B) Real time binding of recombinant hACE2 to immobilized SARS-CoV-2 spike protein on the chip surface with an analyte concentration of 100nM. (C) Binding of 2.5ug of RBDc-CLP vaccine, CLP or blank (buffer) to ACE2 in an ELISA assay. Bars represent the OD_{450nm} mean of duplicates samples with a standard deviation.

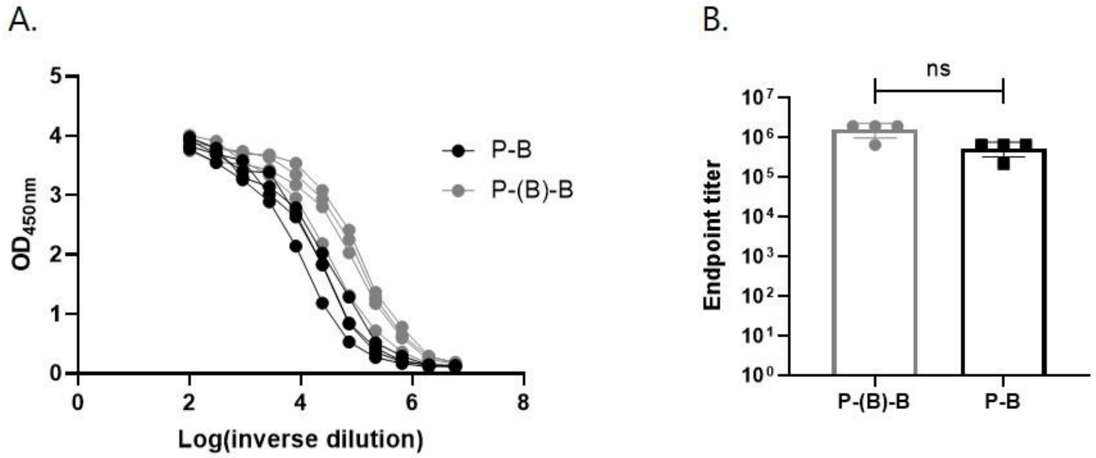

Supplementary figure 3: RBDn-CLP comparison between prime-boost-boost and prime-boost show comparable titers. (A) Dilution curves from ELISA of total anti-SARS-CoV-2 spike (aa35-1227) IgG antibodies detected in sera from BALB/c mice immunized intramuscularly with RBDn-CLP (prime 6.5µg / boost <0.1µg / boost 6.5µg) (n=4) (i.e. P-(B)-B) or RBDn-CLP (prime 5µg / boost 5µg) (n=4) (i.e. P-B). Analyzed sera was obtained two weeks after the boost vaccinations. (B) ELISA results depicted in the form of endpoint titers, using a cut-off 0.2. Bars represent the geometric mean with SD. One sided non-parametric Mann-Whitney test was used for statistical comparison. ns=non-significant, p=0.1143.

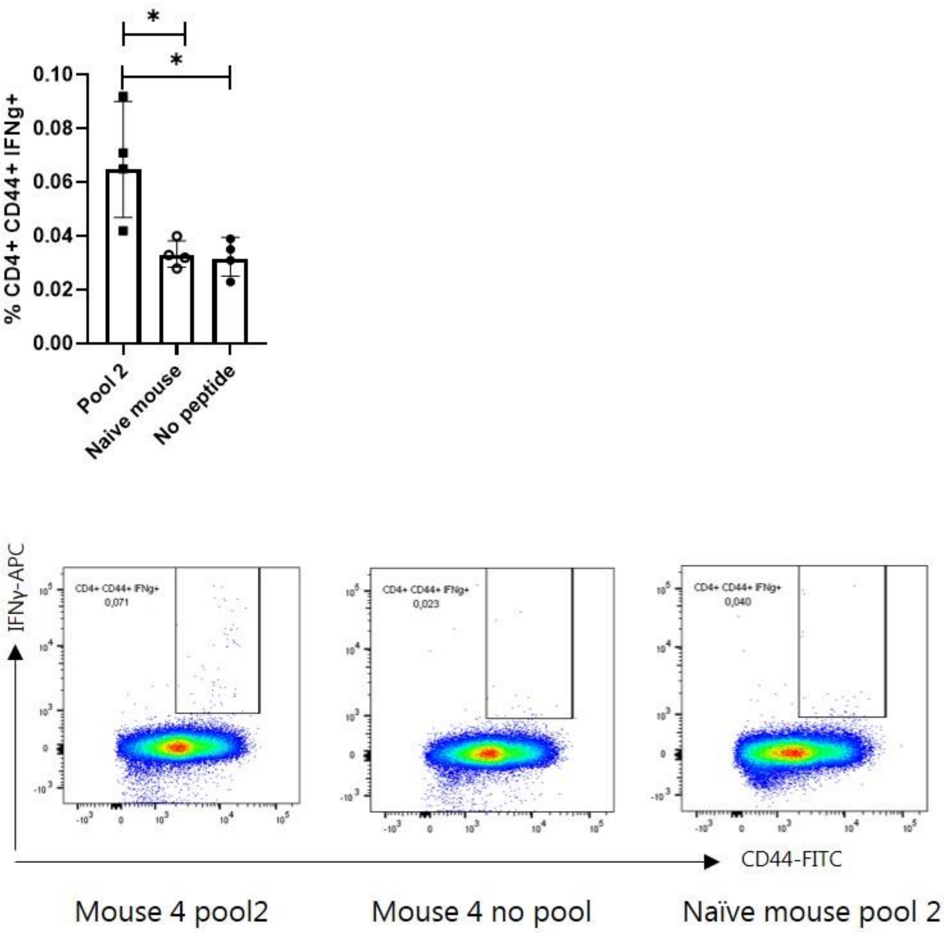

Supplementary figure 4: RBDn-CLP induces a CD4+ T cell response in mice. (A) Percentage of CD4+CD44+IFNg+ cells from mouse splenocytes two weeks after boost immunization (prime 5µg / boost 5µg) with RBDn-CLP (n=4). T cell responses were assessed by flow cytometry after stimulation with a pool of peptides. The peptide pool includes 16-mer peptides with 10 amino acids overlap covering positions 343 to 436 of the SARS-CoV-2 Spike protein. Splenocytes from naïve mice activated with the same peptide pool was used as negative control (naïve) together with unstimulated splenocytes from vaccinated mice (no peptide). Bars represent the mean of the group with a standard deviation. One sided non-parametric Mann-Whitney test was used for statistical comparison. Statistically significant differences are marked by asterisk, *, p=0.0286. (B) Gating strategy to detect activated CD44+ IFNg+ T cells on DIVA software.

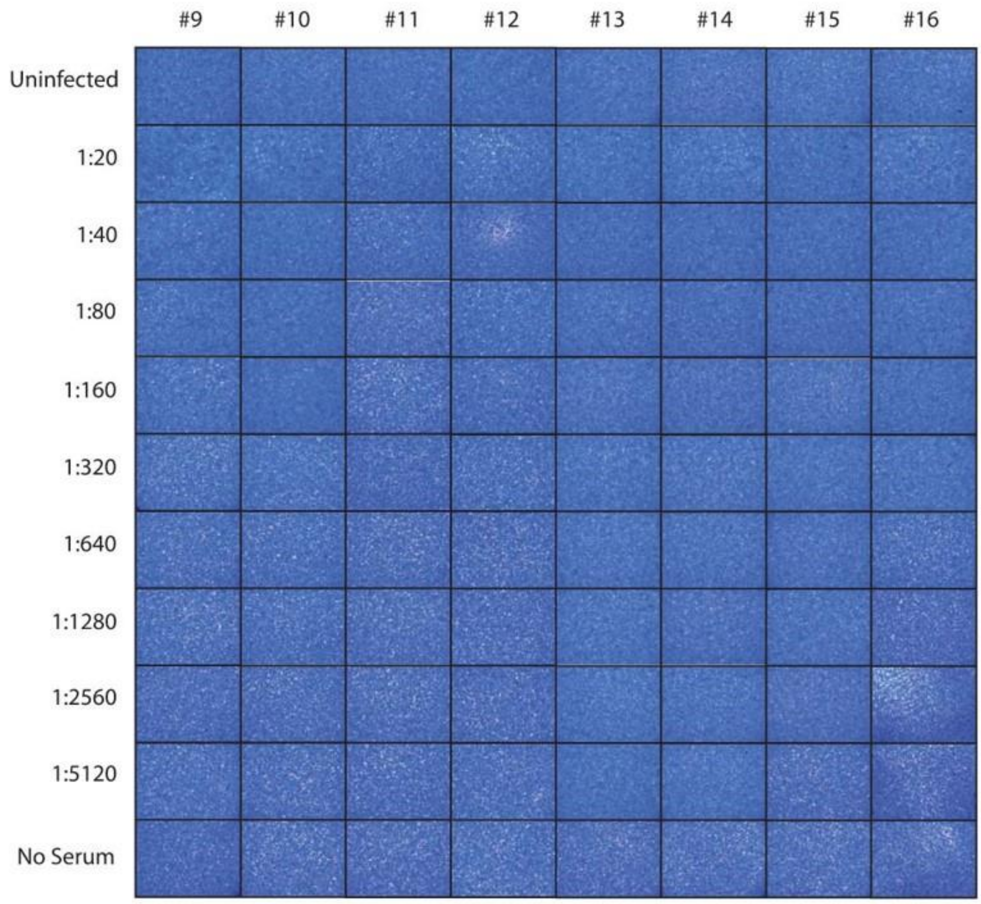

Supplementary figure 5: Sera from mice immunized with RBDc and RBDc-CLP prevent cell entry of SARS-CoV-2 *in vitro*. Microscope images of Vero E6 TWPRSS2 cells infected with 200 TCID50 SARS-CoV-2 pre-incubated with 2-fold serial dilution of serum from mice vaccinated with RBDc (prime 2µg / boost 2µg) (N=4, #9-12) or CLP-displayed RBDc (RBDc-CLP) (prime 1µg / boost 1µg) (n=4, #13-16). Pictures are taken at 4x magnification.

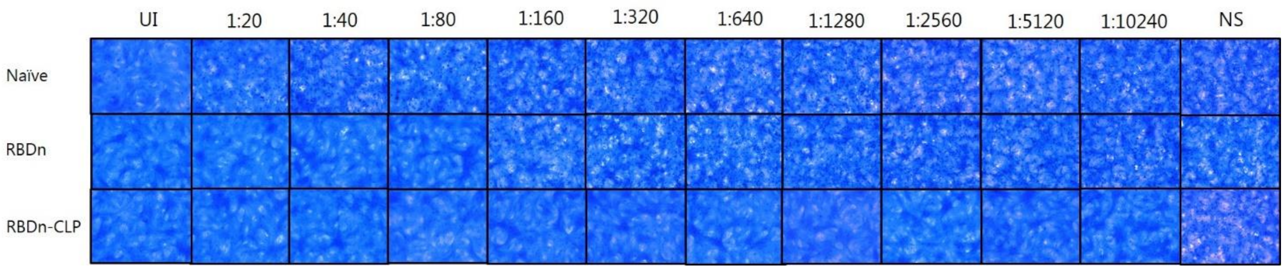

Supplementary figure 6: Sera from mice immunized with RBDn and RBDn-CLP prevent cell entry of SARS-CoV-2 *in vitro.* Microscope images of Vero E6 TWPRSS2 cells infected with 200 TCID50 SARS-CoV-2 pre-incubated with 2-fold serial dilution of serum from mice vaccinated with RBDn-Catcher (prime 5µg / boost 5µg) or CLP-displayed RBDn (RBDn-CLP) (prime 6.5µg / boost <0.1µg / boost 6.5µg). Pictures are taken at 10x magnification.

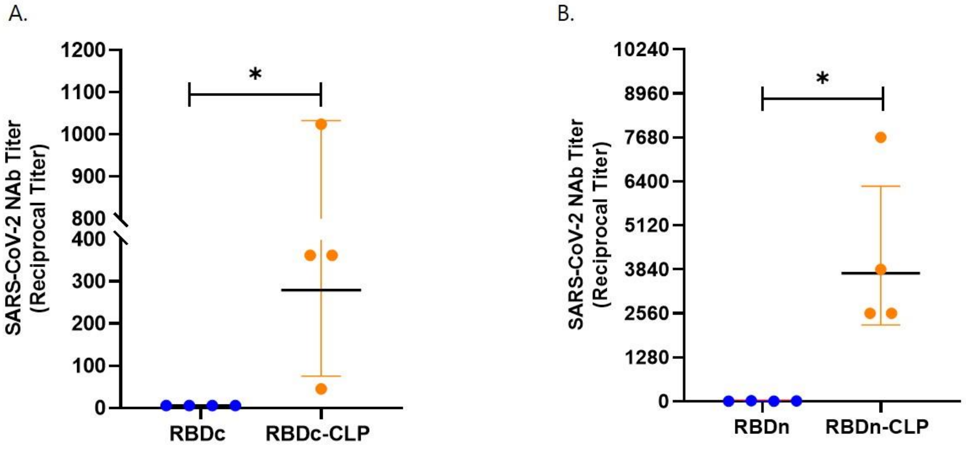

Supplementary figure 7: Serum from mice immunized with RBD-CLP vaccines neutralize SARS-CoV-2 *in vitro.* (A) Serum from mice immunized and boosted with RBDc-CLP (orange) (prime 1µg / boost 1µg) (n=4) or soluble RBDc (blue) (prime 2µg / boost 2µg) (n=4) was mixed with a SARS-CoV-2 virus and tested for cell entry. Each dot represents the reciprocal value of the highest dilution resulting in complete inhibition of virus-induced cytopathogenic effect. Bars represent the geometric mean with SD. One sided non-parametric Mann-Whitney test was used for statistical comparison. A statistically significant differences are marked by the * (p=0.0286). (B) Serum from mice immunized with RBDn-CLP (prime 6.5µg / boost <0.1µg / boost 6.5µg) (orange) (n=4) or soluble RBDn-Catcher (prime 5µg / boost 5µg) (blue) (n=4) was mixed with a SARS-CoV-2 virus and tested for cell entry. Each dot represents the reciprocal value of the highest dilution resulting in complete inhibition of virus-induced cytopathogenic effect. Bars represent the geometric mean with SD. One sided non-parametric Mann-Whitney test was used for statistical comparison. A statistically significant differences are marked by the * (p=0.0286).

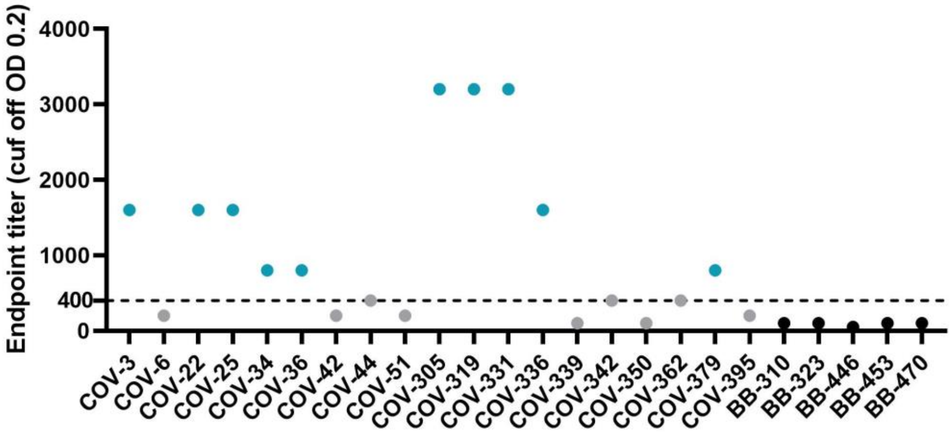

Supplementary figure 8: Total IgG antibody levels measured on human serum from individuals previously recovered from COVID-19. Serum from hospitalized patients that recovered from COVID-19 (COV-xx) (n=19) or serum samples from healthy donors (BB-xxx) (n=4) were measure on ELISA for total IgG against anti-SARS-CoV-2 spike (aa35-1227). A cutoff of set to 400 endpoint titer (dashed line) was used to make a group with high antibody titers (blue) or low antibody titers (grey).
