# Supplementary material for: Capsid-like particles decorated with the SARS-CoV-2 receptor-binding domain elicit strong virus neutralization activity
Source: Nat Commun. 2021 Jan 12;12:324. doi: 10.1038/s41467-020-20251-8 (PMC7804149; doi:10.1038/s41467-020-20251-8)
Supplement: Supplementary file 3 — Reporting Summary [file 41467_2020_20251_MOESM3_ESM.pdf]

nature research

NCOMMS-20-28758A
Corresponding author(s): Adam F. Sander
Last updated by author(s): Nov 12, 2020

Reporting Summary

Nature Research wishes to improve the reproducibility of the work that we publish. This form provides structure for consistency and transparency in reporting. For further information on Nature Research policies, see our Editorial Policies and the Editorial Policy Checklist.

Statistics

For all statistical analyses, confirm that the following items are present in the figure legend, table legend, main text, or Methods section.

n/a	Confirmed	
☐	☒	The exact sample size (*n*) for each experimental group/condition, given as a discrete number and unit of measurement
☐	☒	A statement on whether measurements were taken from distinct samples or whether the same sample was measured repeatedly
☐	☒	The statistical test(s) used AND whether they are one- or two-sided<br>*Only common tests should be described solely by name; describe more complex techniques in the Methods section.*
☒	☐	A description of all covariates tested
☒	☐	A description of any assumptions or corrections, such as tests of normality and adjustment for multiple comparisons
☐	☒	A full description of the statistical parameters including central tendency (e.g. means) or other basic estimates (e.g. regression coefficient) AND variation (e.g. standard deviation) or associated estimates of uncertainty (e.g. confidence intervals)
☐	☒	For null hypothesis testing, the test statistic (e.g. F, t, r) with confidence intervals, effect sizes, degrees of freedom and P value noted<br>*Give P values as exact values whenever suitable.*
☒	☐	For Bayesian analysis, information on the choice of priors and Markov chain Monte Carlo settings
☒	☐	For hierarchical and complex designs, identification of the appropriate level for tests and full reporting of outcomes
☐	☒	Estimates of effect sizes (e.g. Cohen's *d*, Pearson's *r*), indicating how they were calculated

Our web collection on statistics for biologists contains articles on many of the points above.

Software and code

Policy information about availability of computer code

Data collection	DLS data were collected using Wyatt technologies (DynaPro Nanostar, Wyatt technology, Dynamics 7.10.0.21)<br>Attana data were collected using Attache Office 2.1<br>ELISA data were collected on a BioSan HiPo MPP-96 microplate reader, using QuantAssay v0.7.1.4<br>Data on protein purification were collected on Unicorn (version 5.11)<br>Flow cytometry data were collected on DIVA (BD FACSDIVA software v8.0.1)
Data analysis	DLS data were analysed using Wyatt technologies (DynaPro Nanostar, Wyatt technology, Dynamics 7.10.0.21)<br>ELISA and neutralization data were analysed using Graphpad prism (San Diego, version 8.4.3)<br>Flow cytometry data were analysed using Flowjo v10.6.1, and microsoft excel (Microsfot 360, excel 2016) and finally in graphpad prism (San Diego, version 8.4.3).

For manuscripts utilizing custom algorithms or software that are central to the research but not yet described in published literature, software must be made available to editors and reviewers. We strongly encourage code deposition in a community repository (e.g. GitHub). See the Nature Research guidelines for submitting code & software for further information.

Data

Policy information about availability of data

All manuscripts must include a data availability statement. This statement should provide the following information, where applicable:

- Accession codes, unique identifiers, or web links for publicly available datasets
- A list of figures that have associated raw data
- A description of any restrictions on data availability

SARS-CoV-2 spike protein (Sequence ID: QIA20044.1)

Acinetobacter phage AP205 coat protein (Gene ID: 956335) ,

The data that support the findings of this study are available from Bavarian nordic, but restrictions apply to the availability of these data, which were used under license for the current study, and so are not publicly available. Data are however available from the authors upon reasonable request and with permission of Bavarian nordic.

Field-specific reporting

Please select the one below that is the best fit for your research. If you are not sure, read the appropriate sections before making your selection.

- [x] Life sciences
- [ ] Behavioural & social sciences
- [ ] Ecological, evolutionary & environmental sciences

For a reference copy of the document with all sections, see nature.com/documents/nr-reporting-summary-flat.pdf

Life sciences study design

All studies must disclose on these points even when the disclosure is negative.

Sample size

The number of mice used in each study was decided based on previous experience of the minimum number of animals needed to sufficiently detect meaningful differences between groups. (doi: 10.4103/0976-500X.119726)
The number of human serum samples was chosen to have a statistical power in or study, but also to limit ethically the number of samples neeeded. (doi: 10.4103/0974-7788.59946).

Data exclusions

No data were excluded from the study

Replication

Data were perfomed in replicates when possible, or repeated. However when this was not possible, for example in animal studies, the right controls were used to assess the quality of the assay.
figure 1.B : the SDS gel and test coupling was repeated over 5 times, all showed similar results.
figure 2b, d: was repeated 2-3 times and all shown similar results.
Sup figure 1: purification and SDS were repeated at least 5 times and all showed similar results.

Randomization

Randomization for animal studies was not necessary,as there are no possible human bias. Randomization of human serum was done and explained in the section bellow.

Blinding

Randomization for animal studies was not necessary, as there are no possible human bias. Randomization of human serum was done and explained in the section bellow.

Reporting for specific materials, systems and methods

We require information from authors about some types of materials, experimental systems and methods used in many studies. Here, indicate whether each material, system or method listed is relevant to your study. If you are not sure if a list item applies to your research, read the appropriate section before selecting a response.

Materials & experimental systems

n/a	Involved in the study
[ ]	[x] Antibodies
[ ]	[x] Eukaryotic cell lines
[x]	[ ] Palaeontology and archaeology
[ ]	[x] Animals and other organisms
[ ]	[x] Human research participants
[x]	[ ] Clinical data
[x]	[ ] Dual use research of concern

Methods

n/a	Involved in the study
[x]	[ ] ChIP-seq
[ ]	[x] Flow cytometry
[x]	[ ] MRI-based neuroimaging

Antibodies

Antibodies used

Secondary antibodies:
Goat anti-mouse IgG2a, M32207, UC282741, Invitrogen
Goat anti-mouse IgG2b, M32407, TA264696, Invitrogen
Goat anti-mouse IgG3, M32707, RE237181, Thermo Fisher
Goat anti-mouse IgG1, A10551, 1880569, Invitrogen
Goat anti-mouse IgG (H+L), A16072, 70-64-090419, Invitrogen
Rabbit anti-human IgG, P0214, 20043833, Dako

Primary antibodies:

	anti-AP205 Mouse monoclonal antibody (produced in-house)<br>Rat anti-mouse CD4-PE-Cy7, Clone: RM4-5, category: 552775, Lot: 9332915, BD bioscience<br>Rat anti-mouse/human CD44-FITC, clone: IM7, category: 103028, Lot: B262797 Biolegend<br>Rat anti-mouse IFNγ-APC, clone: XMG1.2, category: 505810, lot: B307921, biolegend
Validation	Primary antibodies used for flow cytometry were titrated internally, from previous experiments. Briefly, each primary antibody was diluted down until no signal was detected on positive control cells. The lowest dilution that gave full recognition of our markers was selected. Isotype controls were used to see the background binding of each antibody.

Eukaryotic cell lines

Policy information about cell lines

Cell line source(s)	Aarhus lab: VeroE6-hTMPRSS2 - Kindly provided by Stefan Pöhlmann, university of Göttingen who generated the hTMPRSS2 expressing cell line from VeroE6 (ATCC VERO C1008 [Vero 76, clone E6, Vero E6] (ATCC® CRL-1586™) by retroviral transduction (Hoffmann et al. 2020).<br>Leiden lab: Vero-E6 cells<br>S2 cells: ExpreS2ion Biotechnologies
Authentication	Aarhus lab: No authentication was performed. Cells were obtained directly from the lab who generated the TMPRSS2 expressing cell line.<br>Leiden lab: commercially purchased (ATCC)<br>S2 cells: Proprietary cell line from ExpreS2ion Biotechnologies/ needed no secondary authentication
Mycoplasma contamination	Aarhus lab: The cell line was tested regularly (approx. every 3-4 weeks) and have at all times tested negative for mycoplasma. We use the MycoplasmaCheck service from Eurofins Genomics for all mycoplasma testing (https://www.eurofinsgenomics.eu/en/genotyping-gene-expression/applied-genomics-services/mycoplasmacheck/).<br>Leiden lab: negative for mycoplasma<br>S2 cells: cell line is tested negative for Mycoplasma infection
Commonly misidentified lines (See ICLAC register)	Aarhus lab: VeroE6 cells are not a commonly misdentified lines, thus this is non applicable. However we would like to point out that the VeroE6 cell line is a "Vero76, clone E6", and named VeroE6, but could be confused as Vero76 cells as both are commonly just annotated "Vero cells".<br>Leiden lab: N/A<br>S2 cells: No misidentified eukaryotic cell lines.

Animals and other organisms

Policy information about studies involving animals; ARRIVE guidelines recommended for reporting animal research

Laboratory animals	For all animal experiments, female balb/c mice were obtained from Janvier Labs at age 6-8 weeks and immunized at age 12-14 weeks. Mice were acclimatized for at least 1 week before experiments were performed. Mice were kept in rooms at a temperature of 22oC (±2oC), with a humidity of 55% (±10%), air in the room was changed 8-10 times/hour, according to Danish animal experiments regulations (bekendtgørelse n12 from 07.01.2016).
Wild animals	No wild animals were used in this study
Field-collected samples	No field collected samples were used in this study
Ethics oversight	All animal experiments were conducted in accordance with national Danish guidelines and National Animal Experiments Inspectorate (Dyreforsøgstilsynet, license no. 2018-15-0201-01541). Mice were housed in an AAALAC accredited facility in accordance with good animal practice as defined by FELASA.

Note that full information on the approval of the study protocol must also be provided in the manuscript.

Human research participants

Policy information about studies involving human research participants

Population characteristics	Patient characteristics from a variety of convalescent patients (from asymptomatic to hospitalized patients) are described elsewhere. (Harritshoej, LH et al; Comparison of sixteen serological SARS-CoV-2 immunoassays in sixteen clinical laboratories. MedRxiv, August 02, 2020, DOI:10.1101/2020.07.30.20165373).
Recruitment	The samples from SARS-CoV-2 convalescent individuals were obtained from a variety of convalescent patients in the Capital Region of Denmark with a confirmed SARS-CoV-2 NAAT result: The NAAT results were identified in the Danish Microbiology Database (MiBa) from February 2020 to April 2020. Among 3692 individuals who were randomly contacted via public secure mail 639 persons responded. Blood samples were obtained from each person from 3 - 11th of May 2020. Epidemiologic and clinical data were self-reported in an electronic questionnaire completed on the day of blood sampling. Healthy controls were obtained from archived samples from anonymized blood donors bled before the pandemic, in 2018 to 2019.<br>Samples from 150 individuals bled on May 3rd were included in a national validation study of SARS-CoV-2 antibody immunoassays. Of these, 20 samples were randomly and blindingly selected for our study.<br>We do not expect any bias int the selection of healthy patients or coronavirus positive patients.

Ethics oversight	The study of samples from individuals recovered from Covid-19 infection for validation of serological SARS-CoV-2 assays was approved by the Regional Scientific Committee for the Capital Region of Denmark (H-20028627).<br>Blood donors were asked for consent for using archive samples for their use in the validation of new methods and assay investigations as quality control projects.

Note that full information on the approval of the study protocol must also be provided in the manuscript.

Flow Cytometry

Plots

Confirm that:

- [x] The axis labels state the marker and fluorochrome used (e.g. CD4-FITC).
- [x] The axis scales are clearly visible. Include numbers along axes only for bottom left plot of group (a 'group' is an analysis of identical markers).
- [x] All plots are contour plots with outliers or pseudocolor plots.
- [x] A numerical value for number of cells or percentage (with statistics) is provided.

Methodology

Sample preparation	Spleen from mice were harvested, mashed through a net and resuspended in appropriate buffer
Instrument	Fortessa 3-laser instrument (BD Biosciences)
Software	FlowJo software (Tree Star, Ashland, OR)
Cell population abundance	Cells were not sorted prior to collection, thus representing a standard spleenocyte populations from a healthy mouse.
Gating strategy	Gating was done on fully stained but not activated lymphocytes, to define the negative and positive populations. Cells were gated for single cells (using FSC-FSC-H), then lymphocytes (SSC-FSC), then gated for CD4+, and finally gated for activated IFNg producing cells (CD44+-IFNg+)

- [x] Tick this box to confirm that a figure exemplifying the gating strategy is provided in the Supplementary Information.
